# Prevalence of constipation and associated factors in university hospital inpatients

**DOI:** 10.20407/fmj.2024-006

**Published:** 2024-08-28

**Authors:** Yuka Sano, Junko Sugama, Hiroe Koyanagi, Ryoko Murayama, Takuma Ishihara, Masushi Kohta, Keiko Mano

**Affiliations:** 1 Graduate School of Health Sciences, Division of Medical Sciences, Fujita Health University, Toyoake, Aichi, Japan; 2 Division of Nursing, Fujita Health University Hospital, Toyoake, Aichi, Japan; 3 School of Health Sciences, Faculty of Nursing, Fujita Health University, Toyoake, Aichi, Japan; 4 Research Center for the Implementation of the Nursing Science Initiative, Innovation Promotion Division, Research Promotion Headquarters, Fujita Health University, Toyoake, Aichi, Japan; 5 Innovative and Clinical Research Promotion Center, Gifu University Hospital, Gifu, Gifu, Japan

**Keywords:** Constipation, Defecation care, Defecation difficulty, Nursing record

## Abstract

**Objectives:**

We aimed to determine (1) the prevalence of constipation among inpatients, (2) the prevalence and symptoms of difficult defecation among constipated inpatients, and (3) the factors associated with constipation.

**Methods:**

We performed a retrospective cohort study over a single day at one university hospital. We analyzed the nursing records for inpatients who had been hospitalized for at least 3 days. The survey items included the symptoms associated with defecation difficulty and nutritional intake. The symptoms of difficult defecation were defined as (1) fewer than three spontaneous bowel movements per week; (2) lumpy or hard stools (Bristol stool form scale types 1–2); (3) straining during defecation; and (4) the sensation of incomplete evacuation during defecation, based on the Roma-IV diagnostic criteria. Constipation was defined as the presence of two or more symptoms of defecation difficulty. Univariate and multivariate analyses were performed to determine the constipation status of the patients.

**Results:**

The prevalence of constipation in the university hospital was 12.2%, and the department with the highest prevalence of difficulty with defecation was the Psychiatry Department (64.1%). Of the patients with constipation, 36.8% exhibited symptoms of defecation difficulty other than low frequency of defecation. The factor that was significantly associated with constipation after admission was pre-admission constipation (odds ratio=8.92, *p*<0.01).

**Conclusions:**

Subjective assessment has limitations for the accurate determination of constipation status. In addition, patients with a history of constipation before admission require early interventions to aid defecation following their admission.

## Introduction

Constipation is a defecation disorder that can be classified as organic constipation, involving morphologic changes in the intestinal tract, or functional constipation, resulting from a deficit in intestinal function. It is characterized by unpleasant symptoms, such as abdominal pain, distension, a sensation of incomplete evacuation, pain during defecation, and loss of appetite. The causes include a low level of physical activity, owing to illness, the use of therapeutic drugs, and others. Furthermore, inpatients may develop defecation problems because of changes in their environment and medical examinations. Constipation can lead to various problems, including discomfort, stress on the circulation secondary to straining during defecation, and the risk of bowel obstruction. These problems can result in an extended period of hospitalization, a decrease in quality of life,^1,2^ and high healthcare costs.^3^ The risk of death in patients with constipation has been reported to be higher than in those without this condition.^4^

Nurses should aid the defecation of their patients, in order to promote their ability to focus on their treatment and reduce the physical risks. Common defecation care practice involves nurses obtaining information about their patients’ defecation status from them or their family upon admission and developing an appropriate care plan. However, when a patient’s level of consciousness or condition makes collecting information regarding their bowel movements challenging, nurses often assess the need for defecation care on the basis of defecation frequency.^5,6^ Nevertheless, the lack of a comprehensive defecation assessment when using this approach may hinder effective defecation care.

Sasaki *et al.*^7^ reported that nursing defecation care is often insufficient. They found that hospital nurses tend to prioritize other types of nursing and reported instances where nurses overlooked their patients’ lack of defecation for several days. Owing to the prioritization of medical treatments for nurses, ensuring the provision and evaluation of defecation care for patients has been challenging, particularly for those patients who are seriously ill and unable to communicate their symptoms. This prioritization of medical treatments in nursing detracts from the adequate provision of defecation care and the evaluation of defecation status for inpatients. Previous studies^8–11^ regarding the prevalence of constipation and the associated factors in hospitals have shown prevalences of constipation of 27%–63%. This variation in prevalence could be attributed to differences in the definition of constipation used and the characteristics of the participants, such as their age, dietary habits, and medication.

The factors associated with constipation during hospitalization identified in surveys of inpatients include older age,^12,13^ muscle mass, a history of heart failure,^13^ extended hospitalization, the use of opioids,^12^ and the use of laxatives.^14^ However, few studies have investigated the prevalence of constipation in patients admitted to university hospitals, and there is little high-quality evidence related to this topic.

To reduce the number of inpatients with constipation in university hospitals, the prevalence of constipation and the characteristics of the affected inpatients first need to be determined. Hence, in the present study, we calculated the prevalence of constipation across all the inpatient wards at a university hospital and identified those with a high prevalence of constipation. The secondary objectives were to identify symptoms of difficult defecation in inpatients with constipation and to identify the factors associated with constipation in each ward.

## Methods

### Setting and participants

We performed a retrospective cohort study at a university hospital in Aichi Prefecture, Japan, which has 1,376 beds in 26 departments. The study was performed on a single day in September 2022. The nursing records of the inpatients who had been hospitalized for at least 3 days on this date were studied, and those relating to discharged patients, outpatients, and patients in the central operating room were excluded.

### Variables

The factors related to defecation difficulty, as well as the usage and content of constipation treatments, were recorded. The Roma-IV diagnostic criteria^15^ list four symptoms of difficult defecation: (1) fewer than three spontaneous bowel movements per week; (2) lumpy or hard stools (Bristol stool form scale types 1–2)^16^; (3) straining during defecation; and (4) a sensation of incomplete evacuation during defecation.

The constipation treatments used were medication and/or means of stool evacuation. These treatments included oral medications (including Chinese herbal medicine), such as dilatant laxatives, osmotic laxatives, stimulants, epithelial function-altering drugs, and bile acid transporter inhibitors; suppositories and enemas for external use; and manual maneuvers. The brief use of laxatives for gastrointestinal contrast studies or gastrointestinal fluoroscopy examinations was excluded.

The studied patients were asked to respond using one of three options: yes, no, or unknown. Constipation was defined as the presence of two or more symptoms of defecation difficulty. Evaluations were conducted before and after admission, with the period from admission to the study date defined as the post-admission period. Items not listed in the nursing records were confirmed directly with the study subjects, who were deemed to be lucid by the nurse-in-charge on the day of the survey with reference to the Glasgow Coma Scale (GCS)^17^ in the nursing records.

The patient characteristics collected included the department to which they were admitted, age, and their level of consciousness, according to the GCS. These items were recorded by the charge nurses after reviewing the nursing records of the study subjects.

The principal nutritional factor analyzed was the change in nutrient intake after admission. The changes in the dietary and nutrient intake following admission were assessed, with respondents selecting one of four options: decreased, no change, increased, or missing meals. The survey was administered by charge nurses on the same day, who also noted whether the changes in nutrient intake were associated with enteral or intravenous nutrition. The meals missed owing to participation in the survey were excluded.

### Study procedure

1)  The principal investigator asked the chief nursing officer to distribute the survey to the nurses and obtained consent from the nursing managers. The nursing managers then explained and distributed the survey forms to day-shift nurses.
2)  Nurses who consented to study data collected from the nursing records of patients under their care for at least 3 days post-admission and completed the study forms.
3)  Data were entered into the survey form by the participating nurses.
4)  Completed study forms were placed in special collection bags by day-shift nurses.
5)  Data were collected at the end of the day shift by the person in charge of the study.

### Analysis

**Figure 1:**
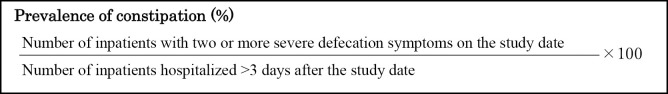
Formula for determining the prevalence of constipation in the inpatients

Figure 1 shows the method used to calculate the prevalence of constipation.

Constipation was defined as a condition in which two or more of the symptoms of defecation difficulty were present. The prevalence of constipation was calculated as the total number of patients with constipation for each department. “Unknown” responses were regarded as indicating a lack of constipation, but missing values were not included.

The factors associated with constipation were identified using univariate and multivariate analyses. In the univariate analysis, descriptive statistics was used to determine constipation status after admission, the factors associated with defecation, and the factors associated with nutrition. Subsequently, the χ^2^ test, Fisher’s exact test, and Mann–Whitney U test were performed as appropriate.

Binomial logistic regression was used for multivariate analysis. The dependent variable was the constipation status after admission. The independent variables were the constipation status; constipation treatment and its components as the factors associated with defecation; dietary changes and nutrient intake as factors associated with nutrition; and age, medical department, and level of consciousness as basic attributes. The significance level was set at 5%, and a *p*-value of <0.05 was considered to indicate statistical significance. SPSS Statistics v.29.0 (IBM Inc., Armonk, NY, USA) was used to perform the analyses. Missing data were included in the analysis.

### Ethical considerations

All procedures that involved human participants were performed in accordance with the ethical standards of the institutional and/or national research committee and with the 1964 Declaration of Helsinki and its later amendments or comparable ethical standards. This study was conducted with the approval of the Fujita Health University Ethics Review Committee (approval no. HM22-090).

## Results

### Characteristics of the patients studied

The study included 932 patients who were hospitalized for >3 days of 1,258 (74.1% of the total) who were in the hospital on the study date. We excluded 311 patients who had been hospitalized for <3 days on the study date and 15 whose nursing records were incomplete.

The characteristics of the patients are presented in Table 1. The prevalence of constipation before admission was 6.1% (57/932). The most common treatment for constipation was laxative medication (17.9%). The median age of the patients was 70 years (interquartile range [IQR]: 52–79). The median GCS score was 15 points (IQR: 15–15). The Gastroenterology ward had the largest number of inpatients with constipation (n=134; 14.4%).

### Prevalence of constipation

Table 2 presents data regarding the prevalence of constipation in the university hospital. The overall prevalence of constipation was 12.2% (n=114). Among the departments with a prevalence of ≥10%, the Psychiatry department had the highest prevalence (n=25; 64.1%), followed by the Ophthalmology department (n=9; 24.3%), the Palliative Medicine department (n=6; 19.4%), the Obstetrics and Gynecology department (n=8; 19.0%), and the Cardiology department (n=9; 17.6%).

### Symptoms and prevalence of defecation difficulty in the patients with constipation

Table 3 lists the symptoms of defecation difficulty reported by the 114 patients with constipation. The symptoms were divided into 11 patterns. Twenty patients (17.5%) reported all four symptoms that constitute the definition of constipation (pattern 1), 28 (24.6%) reported three symptoms (patterns 2–5), and 66 (57.9%) reported two symptoms (patterns 6–11). Of the patients with constipation, 42 (36.8%) exhibited patterns 5, 9, 10, or 11 of symptoms of defecation difficulty other than a lower frequency of defecation.

### Factors associated with constipation

The factors associated with defecation and nutrition are presented in Table 4. When these data were compared with the equivalent data in Table 1, the key difference was that the number of patients who were receiving treatment for constipation had increased from 175 (18.8%) before admission to 289 (31.0%) after admission. The most common treatment for constipation was laxative medication, both before and after admission. The use of constipation treatment after admission (*p*<0.01) was significantly associated with the presence of constipation at the same time. Most of the respondents (n=565; 60.6%) reported no change in their nutrient intake after admission. Following admission, constipation was significantly associated with nutrient intake (*p*<0.01) and the method of nutrient intake (*p*<0.01) at that time.

The results of the binomial logistic regression analysis are presented in Table 5. The Pulmonology department had the lowest prevalence of constipation, but the following departments had a significant prevalence of constipation: Psychiatry (odds ratio [OR]=39.57; *p*<0.01), Pediatrics (OR=19.41; *p*<0.01), Ophthalmology (OR=6.30; *p*=0.01), Obstetrics and Gynecology (OR=6.05; *p*=0.02), and Neurology (OR=5.58; *p*=0.02). Age (OR=1.03; *p*=0.03), GCS score (OR=1.83; *p*=0.04), and constipation before admission (OR=8.92; *p*<0.01) were associated with constipation after admission.

## Discussion

In the present study, we have characterized the prevalence of constipation by department in a university hospital and the symptoms of difficult defecation in patients with constipation, and identified factors associated with constipation in each ward. We made the following three key findings. First, the prevalence of constipation in the university hospital was 12.2%, and the Psychiatry department had the highest prevalence, of 64.1%. Second, of the patients with constipation, 36.8% experienced symptoms of difficult defecation other than a lower frequency of bowel movements. Third, we found that constipation before admission was associated with constipation after admission.

### Prevalence of constipation by department

We believe that the departments that should be prioritized for defecation care intervention are those with an overall prevalence >12.2%, which were the Psychiatry, Ophthalmology, Palliative Medicine, Obstetrics and Gynecology, and Cardiology departments. The prevalence of constipation varied from 3.0% to 64.1%, with the Psychiatry department having the highest prevalence. In previous studies,^18–21^ the prevalence of constipation in psychiatric inpatients was reported by disease rather than for the entire psychiatric inpatient population. The prevalence of constipation by psychiatric disease in inpatients in previous studies was 14.3%–34.2% for depression^20,21^ and 36.3%–36.6% for schizophrenia.^18,19^ Thus, the prevalence of constipation in the psychiatric inpatients in the present study was approximately twice that reported previously. This difference may be attributable to the following: (1) the prevalence of constipation in psychiatric inpatients in the present study included inpatients with diseases other than psychiatric diseases; (2) the study was of inpatients at a university hospital, who may have had more serious conditions, and there may have had a higher prevalence of constipation than those in non-university psychiatry departments; and (3) the criteria used to define constipation in previous studies differed from those used in the present study. Furthermore, the previous studies used a range of criteria for constipation: some used different criteria,^18,21^ one report did not clearly state the criteria used,^20^ and in another one, at least one new prescription medication for constipation was used after admission and during the study period.^19^

Many factors contribute to constipation in psychiatric inpatients, including their treatment (for example, psychotropic medications have anticholinergic effects), exercise and diet, the habitual administration of anti-constipation medications,^22^ and the severity of the psychiatric disease (if severe, patients may be unable to accurately report their symptoms).^23^ Therefore, the reason for the high prevalence of constipation among patients in the Psychiatry department identified in the present study may have been the inclusion of individuals with constipation from other medical departments and the different set of criteria that were used to define constipation.

The prevalence of constipation in the Ophthalmology department was 24.3%. To the best of our knowledge, there have been no previous studies that have demonstrated a prevalence of constipation of approximately 1 in 4 ophthalmology inpatients. However, this relatively high prevalence may have been previously overlooked because such patients are generally independent with respect to their activities of daily living and have shorter hospital stays than patients in other departments.^24^

The factors that contribute to constipation include mydriatic medications (anticholinergics)^25^ and the low level of physical activity during hospitalization.^26^ In particular, ophthalmology patients are often admitted to university hospitals for emergency surgery to treat retinal detachment secondary to trauma or diabetic retinopathy. This low level of physical activity may be the result of positional and rest restrictions, such as the necessity to lie prone or on one’s side for postoperative rest, or autonomic neuropathy secondary to diabetes mellitus.^27^

The other prevalences of constipation were 19.4% in palliative care patients, 19.4% in obstetrics and gynecology patients, and 17.6% in cardiology patients, which are similar to the prevalences identified in previous studies.^28–31^

The Pulmonology department had the lowest prevalence of constipation, of 3.0%. However, this department had the second highest prevalence of constipation treatment after admission (42.9%), following that of the Psychiatry department (48.7%). Constipation can exacerbate chronic obstructive pulmonary disease and asthma,^32,33^ and defecation management through constipation treatment is necessary to prevent the exacerbation of respiratory disease. Therefore, given the effects of constipation on respiratory conditions and the measures that are implemented to prevent it, it is not surprising that the prevalence of constipation in the Pulmonology department was lower than that in the other departments.

Taken together, these findings suggest that the multidisciplinary management of defecation difficulty and the allocation of medical resources should be prioritized for patients in the Psychiatry department, to address the high prevalence of constipation. This multidisciplinary support may involve physicians, nurses, dietitians, pharmacists, and other professionals.

### Symptoms and prevalence of defecation difficulty in patients with constipation

The prevalence of symptoms of defecation difficulty other than a lower frequency of defecation was 36.8%. However, in a previous survey of nurses working in adult hospital wards, most reported that they used a low frequency of defecation as a diagnostic indicator of constipation.^6^ These results suggest that 36.8% of patients with constipation could be missed if a diagnosis of constipation is made solely on the basis of defecation frequency. Therefore, subjective assessments of defecation difficulty alone cannot be used to accurately determine the prevalence of constipation. In the Psychiatry department, patients may be indifferent to defecation or mentally incapable of appropriately reporting this condition, owing to their disease characteristics or the side effects of psychotropic drugs. Therefore, the judgment of constipation status on the basis on GCS would not yield accurate results, and it was difficult to identify constipation according to the level of consciousness of these patients.

For the accurate determination of the prevalence of constipation, we recommend the use of abdominal ultrasonography,^34–36^ which provides both subjective and objective information regarding defecation difficulty and is necessary for effective defecation care. Ultrasonographic images obtained from patients with constipation reveal crescent-shaped, hyperechoic areas that indicate stool retention or hard stool retention, enabling a more objective evaluation.^37^

### Characteristics related to constipation

Typical characteristics of patients with constipation after admission include constipation before admission and an impaired level of consciousness. Patients with constipation before admission should receive defecation care as soon as they are admitted, because early interventions improves quality of life.^1–3^ Therefore, if symptoms of defecation difficulty are identified upon admission, early defecation care should be provided to relieve and manage constipation during hospitalization and the recovery period following discharge.

### Study validity and limitations

The present study was conducted at a university hospital, and we believe the findings should result in the modification of care policies at university hospitals for inpatients requiring advanced care. However, in different hospital settings, such as in rehabilitation or convalescence wards, the backgrounds and habits of the patients should be considered.

The study had several limitations. First, the nursing records lacked detailed information regarding two criteria in the Rome-IV diagnostic criteria (a sensation of anorectal obstruction and the need for manual maneuvers, such as digital evacuation and support of the pelvic floor), which may have affected the calculated prevalence of constipation. Second, variations in GCS score data may have been caused by the collection of responses by a number of different nurses. Third, the subjective responses of patients with GCS score <14 may have low reliability and validity. Fourth, only the patient characteristics and factors related to nutrition were included as factors contributing to constipation, and therefore other factors contributing to constipation risk may have been overlooked. Fifth, “unknown” responses were included in the “no constipation” category. Straining and the sensation of incomplete evacuation on defecation are the typical symptoms reported by patients, but if they could not report problems, owing to the severity of their disease, their status was classified as “unknown,” which may have affected the calculated prevalence of constipation.

### Conclusions

The prevalence of constipation in a university hospital was found to be 12.2%, with the Psychiatry department being the most in need of prioritized defecation care interventions. The prevalence of constipation in the Ophthalmology department was 24.3%, a relatively high prevalence that has not been reported previously. Of the total number of patients with constipation, 36.8% were only evaluated subjectively; therefore, objective criteria based on an ultrasonographic examination should be used in future studies. Patients with constipation before admission require defecation care early during their period of hospitalization.

## Figures and Tables

**Table1:** Characteristics of the patients studied

Characteristic		Total (n=932)
Age		70 (52, 79)
GCS^a^		15 (15, 15)
Constipation before admission	yes	57 (6.1)
Treatment for constipation before admission	yes	175 (18.8)
Departments		
Related departments of gastroenterology^b^		134 (14.4)
Mixed internal medicine^c^		102 (10.9)
Related departments of neurology^d^		93 (10.0)
Pulmonology^e^		84 (9.0)
Orthopedics^f^		74 (7.9)
Mixed surgery^g^		72 (7.7)
Related emergency medicine departments^h^		72 (7.7)
Rehabilitation medicine		59 (6.3)
Cardiology^i^		51 (5.5)
Pediatrics		42 (4.5)
Obstetrics and Gynecology^j^		42 (4.5)
Psychiatry		39 (4.2)
Ophthalmology		37 (4.0)
Palliative medicine		31 (3.3)
Constipation treatment^k^		
Laxative medication	yes	167 (17.9)
Topical (suppository)	yes	5 (0.5)
Topical (enema)	yes	3 (0.3)
Manual maneuvers to facilitate defecation	yes	2 (0.2)
Constipation treatment by department		
Related departments of gastroenterology^b^	yes	34 (25.4)
Mixed internal medicine^c^	yes	35 (34.3)
Related departments of neurology^d^	yes	37 (39.8)
Pulmonology^e^	yes	36 (42.9)
Orthopedics^f^	yes	14 (18.9)
Mixed surgery^g^	yes	23 (31.9)
Related departments of emergency medicine^h^	yes	25 (34.7)
Rehabilitation medicine	yes	25 (42.3)
Cardiology^i^	yes	10 (19.6)
Pediatrics	yes	3 (7.1)
Obstetrics and Gynecology^j^	yes	12 (28.6)
Psychiatry	yes	19 (48.7)
Ophthalmology	yes	4 (10.8)
Palliative medicine	yes	12 (38.7)

Data are median (first quartile, third quartile) or n (%). Missing values are included in the denominator. ^a^ GCS, Glasgow Coma Scale. ^b^ Related departments of gastroenterology include Gastroenterology and Gastroenterologic surgery. ^c^ Mixed internal medicine includes Rheumatology and collagen disease, Hematology and chemotherapy, Nephrology, and Endocrinology and metabolism. ^d^ Related departments of neurology include Neurosurgery, Neurology, and Stroke. ^e^ Pulmonology includes Pulmonology, Allergy, and Pulmonary surgery. ^f^ Orthopedics includes Orthopedic surgery, Plastic surgery, and Spinal surgery. ^g^ Mixed surgery includes Otorhinolaryngology, Dentistry, Oral surgery, Organ transplantation, Endocrine surgery, and Urology. ^h^ Related departments of emergency medicine include General emergency medicine and Emergency medicine. ^i^ Cardiology includes Cardiovascular surgery and Cardiology. ^j^ Obstetrics and Gynecology includes Obstetrics, Gynecology, and Breast surgery. ^k^ Multiple responses

**Table2:** Prevalence of constipation by department

Department	n (n=932)	Constipation (n=114)	Prevalence of constipation (%)
Psychiatry	39	25	64.1
Ophthalmology	37	9	24.3
Palliative medicine	31	6	19.4
Obstetrics and Gynecology^a^	42	8	19
Cardiology^b^	51	9	17.6
Related departments of neurology^c^	93	9	9.7
Orthopedics^d^	74	7	9.5
Mixed internal medicine^e^	102	9	8.8
Related departments of emergency medicine^f^	72	6	8.3
Related departments of gastroenterology^g^	134	11	8.2
Pediatrics	42	3	7.1
Mixed surgery^h^	72	5	6.9
Rehabilitation medicine	59	4	6.8
Pulmonology^i^	84	3	3.6
Total (hospital-wide)	932	114	12.2

^a^ Obstetrics and Gynecology includes Obstetrics, Gynecology, and Breast surgery. ^b^ Cardiology includes Cardiovascular surgery and Cardiology. ^c^ Related departments of neurology include Neurosurgery, Neurology, and Stroke. ^d^ Orthopedics includes Orthopedic surgery, Plastic surgery, and Spinal surgery. ^e^ Mixed internal medicine includes Rheumatology and collagen disease, Hematology and chemotherapy, Nephrology, and Endocrinology and metabolism. ^f^ Related departments of emergency medicine include General emergency medicine and Emergency medicine. ^g^ Related departments of gastroenterology include Gastroenterology and Gastroenterological surgery. ^h^ Mixed surgery includes Otorhinolaryngology, Dentistry, Oral surgery, Organ transplantation, Endocrine surgery, and Urology. ^i^ Pulmonology includes Pulmonology, Allergy, and Pulmonary surgery.

**Table3:** Prevalence and symptoms of defecation difficulty in patients with constipation

Symptoms of defecation difficulty	Pattern											Number of patients
	1	2	3	4	5	6	7	8	9	10	11	
Fewer than 3 spontaneous bowel movements per week	〇	〇	〇	〇		〇	〇	〇				72
Lumpy or hard stools	〇	〇	〇		〇	〇			〇	〇		77
Straining	〇	〇		〇	〇		〇		〇		〇	75
Sensation of incomplete evacuation on defecation	〇		〇	〇	〇			〇		〇	〇	72
Number of patients	20	6	6	7	9	20	6	7	10	6	17	114
Percentage of respondents (%)	(17.5)	(5.3)	(5.3)	(6.1)	(7.9)	(17.5)	(5.3)	(6.1)	(8.8)	(5.3)	(14.9)	

**Table4:** Relationships between measures related to constipation

Measure		Total (n=932)	Constipation (n=114)	No constipation (n=818)	*P*-value
Constipation treatment after admission	yes	289 (31.0)	65 (57.0)	224 (27.4)	<0.01^a^
Constipation treatment^c^					
Laxative medication	yes	274 (29.4)	58 (50.9)	216 (26.4)	<0.01^b^
Topical (suppository)	yes	10 (1.1)	4 (3.5)	6 (0.7)	0.25^b^
Topical (enema)	yes	8 (1.0)	5 (4.4)	3 (0.3)	<0.01^b^
Manual maneuvers to facilitate defecation	yes	19 (2.0)	8 (7.0)	11 (1.4)	<0.01^b^
Nutrient intake after admission^d^					
Decrease		192 (20.6)	30 (26.3)	162 (19.8)	<0.01^b^
No change		565 (60.6)	62 (54.4)	503 (61.5)	
Increase		45 (4.8)	17 (14.9)	28 (3.4)	
Missing meals		90 (9.6)	4 (3.5)	86 (10.5)	
Method of nutrient intake^d^					
Enteral nutrition		802 (86.1)	109 (95.6)	693 (84.7)	<0.01^b^
Intravenous nutrition		90 (9.7)	4 (3.5)	86 (10.5)	

Data are n (%). ^a^ Chi-square test. ^b^ Fisher’s test or chi-square test. ^c^ Multiple responses to constipation treatment status are included. ^d^ Missing values are included in the denominator. n=40.

**Table5:** Results of the multivariate logistic regression analysis of the factors associated with constipation

Factor	Odds ratio (95% CI)	*P*-value
Age	1.03 (1.00–1.04)	0.03
GCS^a^	1.83 (1.04–3.23)	0.04
Constipation before admission (0: no, 1: yes)	8.92 (4.43–17.96)	<0.01
Method of nutrent intake		
Enteral nutrition	Reference	
Intravenous nutrition	0.55 (0.17–1.77)	0.32
Treatment for constipation before admission (0: no, 1: yes)	1.35 (0.76–2.39)	0.31
Departments		
Pulmonology^b^	Reference	
Psychiatry	39.57 (9.46–165.57)	<0.01
Pediatrics	19.41 (2.56–147.11)	<0.01
Ophthalmology	6.30 (1.50–26.49)	0.01
Obstetrics and Gynecology^c^	6.05 (1.36–26.91)	0.02
Palliative medicine	4.53 (0.76–26.88)	0.10
Cardiology^d^	4.04 (0.96–17.04)	0.06
Related departments of neurology^e^	5.58 (1.38–22.63)	0.02
Orthopedics^f^	2.72 (0.62–11.84)	0.18
Related departments of emergency medicine^g^	2.50 (0.54–11.61)	0.24
Related departments of gastroenterology^h^	2.96 (0.75–11.76)	0.12
Mixed internal medicine^i^	2.29 (0.55–9.47)	0.25
Mixed surgery^j^	1.77 (0.37–8.54)	0.48
Rehabilitation medicine	1.64 (0.31–8.51)	0.55

^a^ GCS, Glasgow Coma Scale. ^b^ Pulmonology includes Pulmonology, Allergy, and Pulmonary surgery. ^c^ Obstetrics and Gynecology includes Obstetrics, Gynecology, and Breast surgery. ^d^ Cardiology includes Cardiovascular surgery and Cardiology. ^e^ Related departments of neurology include Neurosurgery, Neurology, and Stroke. ^f^ Orthopedics includes Orthopedic surgery, Plastic surgery, and Spinal surgery. ^g^ Related departments of emergency include General emergency medicine and Emergency medicine. ^h^ Related departments of gastroenterology include Gastroenterology and Gastroenterological surgery. ^i^ Mixed internal medicine includes Rheumatology and collagen disease, Hematology and chemotherapy, Nephrology, and Endocrinology and metabolism. ^j^ Mixed surgery includes Otorhinolaryngology, Dentistry, Oral surgery, Organ transplantation, Endocrine surgery, and Urology.
